# A highly resolved integrated transcriptomic atlas of human breast cancers

**DOI:** 10.1101/2025.03.13.643025

**Published:** 2025-05-03

**Authors:** Andrew Chen, Lina Kroehling, Christina S. Ennis, Gerald V. Denis, Stefano Monti

**Affiliations:** 1 Section of Computational Biomedicine, Boston University Chobanian and Avesidian School of Medicine, Boston, MA 02118, USA.; 2 Bioinformatics Program, Center for Computing & Data Sciences, Boston University, Boston, MA 022158, USA.; 3 Boston University-Boston Medical Center Cancer Center, Boston University Chobanian and Avesidian School of Medicine, Boston, MA, 02118, USA; 4 Section of Hematology and Medical Oncology, Department of Medicine, Boston University Chobanian and Avesidian School of Medicine and Boston Medical Center, Boston, MA, 02118, USA; 5 Department of Pharmacology and Experimental Therapeutics, Boston University Chobanian and Avesidian School of Medicine, Boston, MA, 02118, USA; 6 Department of Biostatistics, Boston University School of Public Health, Boston, MA 02118, USA.

## Abstract

In this study, we developed an integrated single cell transcriptomic (scRNAseq) atlas of human breast cancer (BC), the largest resource of its kind, totaling > 600,000 cells across 138 patients. Rigorous integration and annotation of publicly available scRNAseq data enabled a highly resolved characterization of epithelial, immune, and stromal heterogeneity within the tumor microenvironment (TME). Within the immune compartment we were able to characterize heterogeneity of CD4, CD8 T cells and macrophage subpopulations. Within the stromal compartment, subpopulations of endothelial cells (ECs) and cancer associated fibroblasts (CAFs) were resolved. Within the cancer epithelial compartment, we characterized the functional heterogeneity of cells across the axes of stemness, epithelial-mesenchymal plasticity, and canonical cancer pathways. Across all subpopulations observed in the TME, we performed a multi-resolution survival analysis to identify epithelial cell states and immune cell types which conferred a survival advantage in both The Cancer Genome Atlas (TCGA) and METABRIC. We also identified robust associations between TME composition and clinical phenotypes such as tumor subtype and grade that were not discernible when the analysis was limited to individual datasets, highlighting the need for atlas-based analyses. This atlas represents a valuable resource for further high-resolution analyses of TME heterogeneity within BC.

## Introduction

Breast cancer (BC) is the most prevalent cancer and the second most common cause of cancer death in women ^1^. It is defined as a malignancy of the epithelial duct in breast tissue, and is a highly heterogeneous disease ^2,3^ for which clinical outcomes and responsiveness to treatment depend greatly on intrinsic factors related to the cancer cell, as well as extrinsic factors related to altered states of immune and stromal cells in the tumor microenvironment (TME) ^4–6^. To improve our understanding of breast cancer progression and treatment, an extensive characterization of the heterogeneity in BC cancer cells and the surrounding tumor microenvironment is required.

Single cell transcriptomics (scRNAseq) remains the method of choice to characterize heterogeneity in tissue composition in terms of cell *types* – categories of cells that perform consistent functions, and in terms of cell *states* – categories of transient functions that can be shared across different cell types ^7,8^. The technology has been extensively applied to profile healthy breast tissue, as part of the larger human cell atlas effort. There are now highly-resolved scRNAseq atlases that have annotated 700,000–800,000 cells across 55–126 samples yielding new insights into the diversity of immune, stromal, and epithelial populations in healthy breast tissue and how these are linked to clinical metadata ^9,10^. However, the application of scRNAseq to profile breast cancer (BC) samples has been limited by cohort sizes, with most studies having fewer than 30 samples ^11–20^. While these individual studies have laid substantial groundwork in characterizing tumor heterogeneity within BC, their limited sample size poses a challenge with reproducibility and identifying statistically significant associations between tissue composition and clinical metadata.

**Figure 1.**
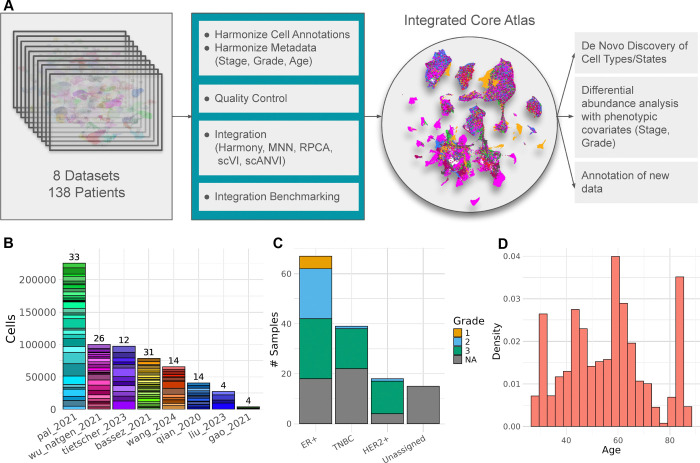
Construction and composition of the integrated breast cancer atlas. A) Overview of the construction of the integrated atlas and downstream analysis. B) Distribution of patients and number of cells per patient sequenced across each of the eight datasets. C) Distribution of tumor subtype and grade information. D) Distribution of ages for all patient donors.

To address this challenge, we integrated eight publicly available scRNAseq datasets of untreated and unsorted BC samples ^11–20^ to create an atlas of transcriptional heterogeneity in BC. The analysis of this combined atlas enabled 1) the reconciliation of mislabeled cell types, 2) the identification of novel cell types, and 3) the association of changes in TME heterogeneity with clinical phenotypes such as tumor subtype and grade with increased statistical power (Fig 1a). Another study has attempted to integrate publicly-available scRNAseq BC data to create an atlas but compared to Xu et al. our study only contains unsorted BC samples, allowing for an unbiased characterization of inter-patient heterogeneity, and our atlas has a much a larger sample size (> 600,000 cells), enabling more robust identification of cell types and differentially abundant populations across phenotypes ^21^.

This integrated scRNAseq atlas provides an unbiased reference landscape of transcriptional heterogeneity in BC, comprised of more than 600,000 cells across 138 patients, the largest of its kind, and represents a valuable resource for hypothesis-driven analysis of TME heterogeneity. The data and methods described herein provide a robust framework for updating this BC atlas as new datasets are made publicly available ^22^.

## Results

### Constructing the integrated core atlas

**Figure 2.**
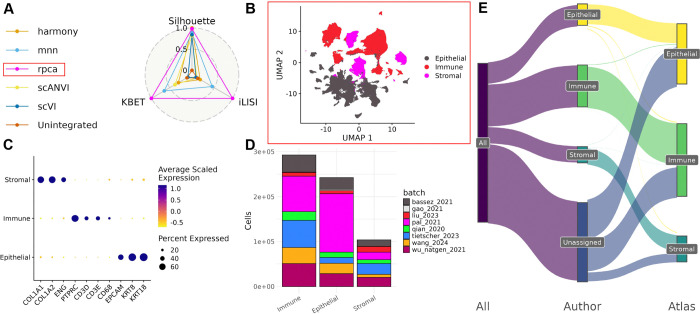
Compartment specific analysis of atlas. A) Batch correction metrics for each integration method. B) UMAP of embeddings from chosen integration method (RPCA) and the separation by broad cell type. C) Expression of canonical markers for each of the broad cell types separated from the atlas. D) Distribution of cells from each dataset in each of the three broad compartments. E) Comparison of broad cell type label between author annotations and atlas-based annotations. F) Expression of canonical markers for the relabeled cells if categorized by the atlas labels. G) Expression of canonical markers for the relabeled cells if categorized by their original labels.

The BC atlas was constructed by integrating eight publicly available scRNAseq datasets of untreated primary BC tumors. Across the eight datasets, samples were sequenced at varying throughput levels, and had different distributions of clinical and patient metadata including subtype, grade, and age (Fig. 1b,c,d). Before integration, batch effects were evident as cells separated by dataset of origin, rather than by biological factors (Supp Fig. 1). Six different integration approaches were applied and the best-performing method, as determined by atlas integration batch correction metrics ^23^ was Seurat’s reciprocal PCA method (RPCA) (Fig 2a).

### Relabeling of broad cell types

To annotate cells in the integrated atlas, we first defined three compartments: epithelial, immune, and stromal, by cross-referencing annotations from SingleR, CellTypist, and author annotations with broad clustering (Louvain clustering with resolution 0.1) (Supp Fig. 2). The resulting compartments showed distinct expression of canonical marker genes (Epithelial: *EPCAM, KRT8, KRT18*; Immune: *PTPRC, CD3D, CD3E, CD68*; Stromal: *COL1A1, COL1A2, ENG*) and diverse representation across all eight source datasets (Fig. 2b,c,d). More than 300,000 cells that previously had no available cell type annotation were annotated using the cluster-based annotations from the atlas (Fig. 2e).

These annotations of broad cell types based on integrated data also resulted in a relabeling of broad cell types for over 9172 cells (around 1% of the atlas) across 5 datasets (e.g., relabeling a cell as stromal that was originally labeled immune).

### Cancer Epithelial Diversity

**Figure 3.**
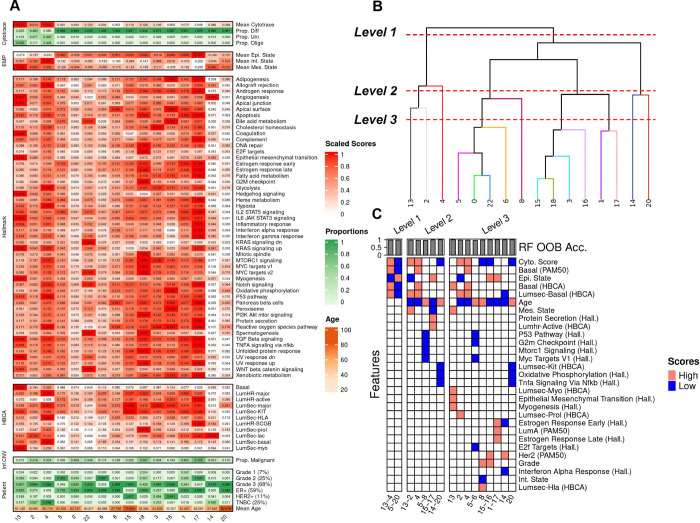
Multi-resolution characterization of Cancer Epithelial Compartment. A) Heatmap of patient metadata, malignancy data (InferCNV), annotation data (HBCA), cell potency data (Cytotrace), plasticity data (EMP), and expression of hallmark pathways (Hallmark). B) Dendrogram of epithelial clusters learned from K2 Taxonomer. C) Top 5 important features, as determined by gini-based importance, from random forest models trained to differentiate different clades within each level of the dendrogram. Red cells indicate that scores related to that feature were higher than the mean in that subset of the dataset.

Intra-tumor heterogeneity within cancer cells stems from multiple factors including but not limited to genomic instability, epigenetic alterations, and clonal evolution which are all implicated in the treatment of cancer ^24^. In this BC atlas, we sought to characterize intra and inter-tumor transcriptional heterogeneity by scoring each cancer cell with respect to potency/stemness ^25^, epithelial-mesenchymal plasticity (EMP) ^26^, malignancy ^27^, ‘activity’ of hallmark pathways ^28^, expression of epithelial cell type markers from HBCA ^10^, as well as clinical metadata related to age, tumor grade, and subtype (Fig. 3a). Further, we sought to find the driving factors of variation by leveraging a recursive partitioning tree-based method *K2 Taxonomer* ^29^ to identify meta-clusters and hierarchical relationships between them (Fig. 3b), and also trained random forests to extract the most discriminative features distinguishing each cluster and meta-cluster (Fig. 3c).

To identify functional groups of cancer cells shared across patients in the atlas, we performed unsupervised clustering (Louvain clustering with resolution 0.1) of cancer epithelial cells and identified 23 clusters that largely stratified by cell typist annotation based on the healthy Human Breast Cell Atlas (HBCA), author subtype, and Pam50 inferred subtype (Supp Fig. 3a,b,c,d). The differentially expressed genes for each epithelial cluster were identified using MAST (Supp Table 1). Several resolution parameters were evaluated (0.1–1.0) with even the coarsest resolution of 0.1 yielding multiple clusters (7,9,10,11,12,19,21) mostly consisting of cells from individual patients, as also revealed by their lowest entropy of donor proportions (Supp Fig. 3e,f). This suggests that the variation captured within these clusters was mostly driven by tumor-specific clonal expansion and, given our goal of defining shared programs of cancer epithelial heterogeneity, they were excluded from further downstream characterization.

#### Multi-resolution characterization of cancer epithelial cells

Among the remaining 16 epithelial clusters we identified the driving factors of variation within our cancer epithelial compartment by training random forest based classifiers at three separate levels of taxonomic resolution (Fig. 3b,c). In previous integrated single cell studies of breast cancer, cancer epithelial heterogeneity was characterized with respect to pam50 and clinical subtypes^11,12^, as well as functional annotations by, e.g., Hallmark signatures ^21^, but has not yet been done in a hierarchical manner to identify the driving factors of variation.

This multi-level characterization revealed segregation of cancer epithelial cells at the highest level (Level 1) by stemness, EMP, and basal phenotypes, with clusters 13 through 4 (13~4) on the right side of the dendrogram exhibiting more stemness, more EMP (more mesenchymal), and being more basal-like compared to clusters on the left side (clusters 0~16). At the next level of the dendrogram (Level 2), cancer cells on both sides segregated by age, with the left branch of the tree (clusters 0~16) segregating by older donors (clusters 0~20, 8, and 15~16) and younger donors (clusters 1~5), and the right branch of the tree (clusters 13~4) segregating by older (cluster 4) and younger (clusters 13~2) patients. At the next level of characterization (Level 3), finer groups of cancer epithelial heterogeneity emerge with clusters exhibiting decreased mitotic spindle activity (cluster 0), decreased TNFA signaling (cluster 20), increased EMT activity (cluster 13), as well as subtype-specific clustering (Luminal A: 1~17, 18; Luminal B: 6, 5, 15, 16; HER2: 3, 14), and grade specific clustering (high grade tumors: 8; low grade tumors: 6, 5, 18).

### Immune and Stromal Cell Diversity in the TME

**Figure 4.**
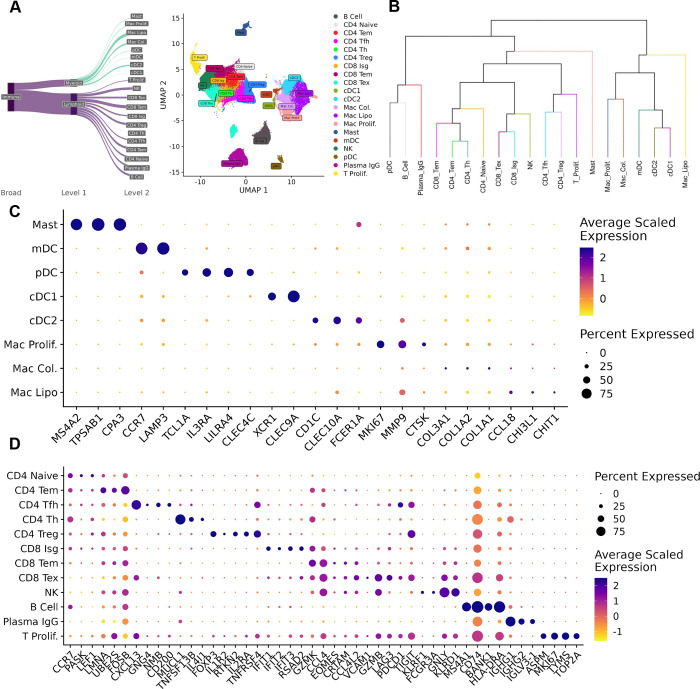
Diversity of immune cell types in the TME. A) Sankey diagram of all immune subtypes characterized in the atlas. B) Dendrogram of immune annotations learned from K2 Taxonomer. C) Dot plot of markers for the lymphoid subtypes identified in the atlas. D) Dot plot of markers for the myeloid subtypes identified in the atlas.

**Figure 5.**
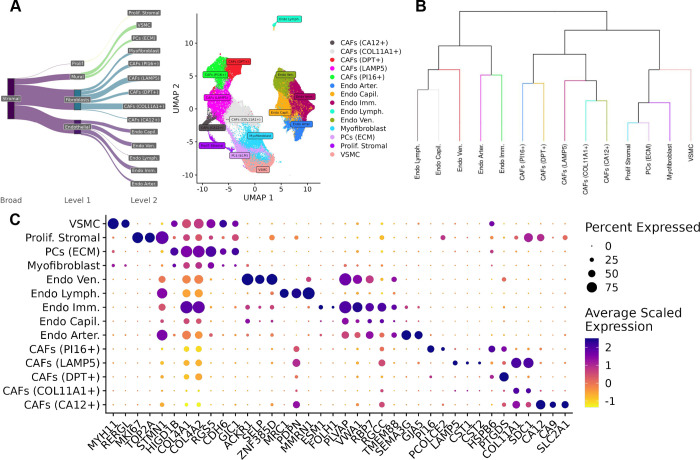
Diversity of stromal cell types in the TME. A) Sankey diagram of all stromal subtypes characterized in the atlas. B) Dendrogram of stromal annotations learned from K2 Taxonomer. C) Dot plot of markers for the stromal subtypes identified in the atlas.

Across the immune and stromal compartments of the atlas, 20 immune and 14 stromal subpopulations were characterized (Fig. 4a,5a). These subpopulations were identified by integrating data from automatic annotation methods: SingleR ^30^ and CellTypist ^31^ (Supp Fig. 4,5), hierarchical tree-based methods (K2 Taxonomer) ^29^ (Fig. 4b, 5b), as well as marker genes obtained from differential expression analysis (MAST) ^32^ (Supp Table 2,3).

#### Reconciliation of immune cell subtypes to pan-cancer subtypes

Macrophages formed three clusters, which were all found to have analogs in previous pan-cancer and breast cancer specific characterizations of myeloid cells ^10,33^. These included a cluster of lipid-associated macrophages *Mac. Lipo* defined by high expression of genes associated with lipid transport and metabolism (*LIPA, LPL, APOE, APOC1*); a cluster characterized by high expression of *MIK67* and *TOP2A* indicating a more proliferative state (*Mac Prolif)*; and a cluster of macrophages *Mac. Col* defined by strong expression of collagen genes (*COL1A1, COL1A2, COL3A1*), a subtype that has been previously been connected to ECM remodeling ^34^ (Fig. 4a,b,c).

T cells formed nine clusters (Fig. 4a), which were also found to have analogs in previous pan-cancer studies ^13,35,36^. These included CD4 and CD8 effector memory T cells (*CD4/CD8 Tem*), which are characterized by expression of *LMNA, FOS,* and *GZMK, EOMES* respectively; CD4 Tregs defined by expression of *FOXP3, IL2RA,* and *TNFRSF4*; an interferon stimulated group of CD8 T cells (*CD8 ISG*) defined by strong expression of interferon signaling genes *IFIT1, IFIT2, IFIT3*; naïve CD4 cells characterized by expression of *CCR7, PASK, LEF1*; exhausted CD4 T cells (*CD4 Ex*) defined by expression of canonical exhaustion markers such as *LAG3, HAVCR2, PDCD1* and *TIGIT*; CD4 T follicular helper cells (*CD4 Tfh*) expressing transcription factors *TOX, TOX2* and chemokine *CXCL13*; CD4 T helper cells (*CD4 Th*) which in comparison to *CD4 Tfh* cells expressed high levels of interleukin genes *IL7R*, as well as the chemokine receptor *CCR6*. Similar to the macrophages, a proliferative T cell cluster (*T. Prolif*) was identified exhibiting strong expression of *MIK67* and *STMN1* (Fig. 4,a,b,d).

NK cells formed one cluster (Fig. 4a,d), which was defined by expression of Killer Cell Lectin receptors (*KLRD1, KLRF1*) and growth factor *FGFBP2*. A previous single cell breast cancer atlas identified 6 NK subtypes, although their atlas only contained 3720 NK cells across all studies, whereas our study identified one cluster with no discernable functional differences across a population of 23508 NK cells. This discrepancy may be attributed to the difference in sample size and clustering strategies between the two atlases ^37^.

Mast cells formed one cluster (Fig. 4a,c), defined by expression of the enzymes *TPSB2, CPA3,* and *TPSAB1*.

B Cells also formed a single cluster defined by canonical B cell markers such as *CD19*, *MS4A1*, *CD74* as well as expression of human leukocyte antigen genes such as *HLA-DRA* and *HLA-DPB1* (Fig. 4a,d).

#### Identification of dendritic subtypes

Dendritic cells formed four clusters (Fig. 4a,c), which include two conventional dendritic cell populations, one characterized by expression of *CLEC9A* (*cDC1*), and another characterized by expression of *CD1C* and *CLEC10A* (*cDC2*); a mature dendritic cell population (*mDC*) defined by *CCL22*, *CCR7*, and *CCL19* expression; and a plasmacytoid dendritic cell population (*pDC*) defined by expression of *GZMB*, *JCHAIN*, and *PTGDS*.

#### Reconciliation of stromal cell subtypes to pan-cancer subtypes

Fibroblasts formed six clusters (Fig. 5a,b,c), which were also reconciled to previously characterized pan-cancer stromal subtypes ^38^. This includes five clusters of cancer-associated fibroblasts (CAFs) consisting of a population of *COL11A1+ CAFs* (*COL11A1*, *COL8A2*) implicated in collagen metabolism ^38^, a population of *LAMP5 CAFs* defined by expression of *LAMP5* and cystatins *CST1* and *CST2* implicated in promoting EMT ^38^, a population of PI16+ CAFs defined by expression of *PI16* and *PCOLCE2*, a population of DPT+ CAFs defined by expression of *DPT* and *CAPN6*, as well as population of CA12+ CAFs defined by expression of *CA12* and *SLC2A1* which has been implicated in glycolysis metabolism and hypoxia ^38^. Apart from these CAFs subtypes, myofibroblasts also formed one cluster by expression of *RGS5* and *CDH6*.

**Figure 6.**
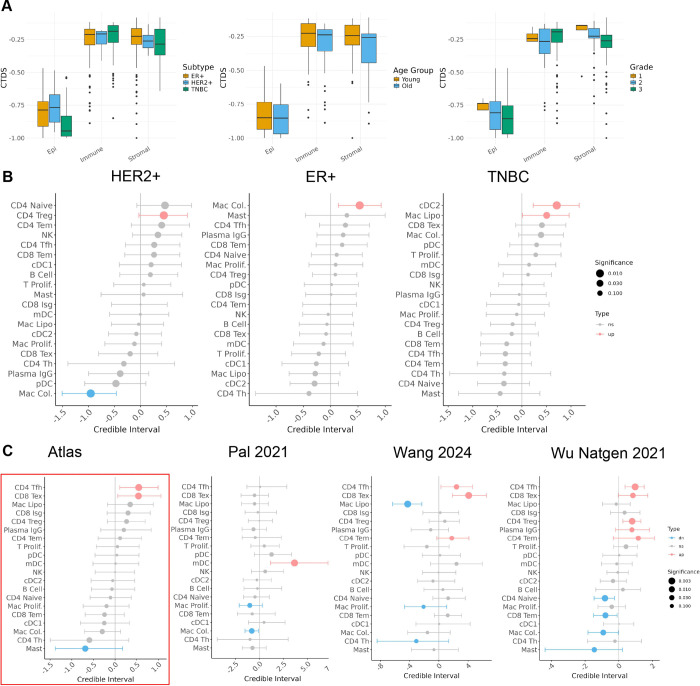
Differential cell type diversity and abundance analysis. A) Cell Type Diversity Score (CTDS) across tumor subtype, patient age, and tumor grade. B) Differential cell type estimates within the immune compartment between HER2+, ER+ and TNBC tumors. B) Differential cell type estimates within the immune compartment between high and low grade tumors.

Endothelial cells (ECs) surround blood vessels in the TME which are responsible for angiogenesis and tumor growth and formed a total of five clusters in the atlas (Fig. 6a). Four clusters emerged based on the function of these endothelial cells in supporting vasculature: endothelial vein, arterial, capillary, and lymphatic cells segregated based on expression of different marker genes (Fig 5a,b,c). A further cluster of immature endothelial cells (*Endo Imm*), defined by expression of *PLVAP, VWA1*, and *CA4*, was identified and has been implicated in poor tumor prognosis ^38^.

Other stromal subtypes captured in the atlas include vascular smooth muscle cells (*VSMC*) defined by expression of *RERGL*, and *MYH11*, extracellular matrix related pericytes (*ECM PCs*) defined by expression of collagen genes *COL4A1*, and *COL4A2*, as well as a set of proliferating stromal cells (*Prolif. Stromal)* characterized by high expression of proliferative markers including *TOP2A*, *MKI67*.

#### Taxonomic characterization of immune and stromal subtypes

We utilized K2Taxonomer to organize the clusters in the immune and stromal compartments into hierarchical taxonomies. Annotating groups of clusters, or clades, mitigates the uncertainty in annotation due to over-clustering. Taxonomic characterization also captures groups of cells sharing similar functions and cell states rather than just cell types.

In the immune compartment, cells largely segregated by lymphoid and myeloid lineages. The exceptions being a population of *pDCs*, which clustered with plasma cells in a clade due to their shared expression of *MZB1* ^39^, and Mast cells, which clustered within the lymphoid clade due to the lack of expression of macrophage and dendritic markers (*CD68*, *CD1C*). These exceptions suggest that immune cells in the TME may share similar transcriptional states despite having different lineages (Fig. 4b).

In the stromal compartment, cells partitioned by endothelial and non-endothelial at the first level, and within the non-endothelial clade, further separated between CAFs and mural cells, and lastly between smooth muscle cells, pericytes, and myofibroblasts, indicating strong transcriptome-based partitioning of these stromal subtypes (Fig. 5b).

### Cell type diversity of tumors changes across phenotypes

Using these improved annotations within the atlas, we used an entropy-based cell type diversity score (CTDS) ^40^ to assess how the overall diversity of the TME changes across patient phenotypes (Fig. 6a). The CTDS score yields the highest value when all cell types (or states) have equal representation in a sample, and the lowest value when a single cell type (or state) is represented in a sample.

Across clinical subtypes, the CTDS of observed epithelial cell states and stromal cell types observed was lowest in TNBC compared to ER+ and HER2+, whereas the average CTDS of immune cell types was highest in TNBC. Across age groups, we observed a consistent decrease in CTDS across all three compartments in older tumors compared to younger tumors. This expands upon previous studies that identified decreased immune cell type diversity in old subjects compared to young subjects, but only within PBMCs ^40^. As tumor grade increased, we also observed a consistent decrease in cell type diversity within the epithelial and stromal compartment. The cell type diversity scores provide a global summary of changes in tumor heterogeneity that can complement the more granular differential abundance analysis of individual cell types within the tumor.

### Improved estimates of differential immune cell type abundance across phenotypes

Using these improved annotations within the atlas, we used *sccomp* ^41^ to estimate differential immune cell type abundance across phenotypes such as tumor grade and subtype (Methods).

HER2+ tumors are typically considered to be ‘hot’ tumors with increased immune infiltration compared to other subtypes of tumors ^42^, but the details of their cell type composition vis-à-vis other tumor subtypes is unclear. In our analysis, we observed that one specific subset of T cells, *CD4 Tregs*, increased in abundance in HER2+ tumors relative to other tumor types, whereas one subset of macrophages, *Mac Col.*, decreased in relative abundance (Fig. 6b).

The immune microenvironment of ER+ tumors have been characterized as macrophage-driven, but the exact subtypes of macrophages driving this microenvironment remain unknown ^43^. In our analysis, we observed that one subset of macrophages, *Mac Col.*, was enriched in ER+ tumors compared to other tumors (Fig. 6b).

TNBC tumors are known to have a heterogeneous immune microenvironment with tumors exhibiting both increased tumor infiltrating lymphocytes (TILS) ^44^, and increased tumor associated macrophages ^45^. However, the exact nature of which immune cell types are enriched has not yet been characterized in studies with adequate statistical power. In our analysis we found that *Mac Lipo.* were enriched in TNBC tumors, as well as a subpopulation of *CLEC10A* conventional dendritic cells (*cDC2).* Previous studies have found that pDCs are expanded ^46^ and that mast cells are be depleted in TNBC tumors ^47^. However, although these specific populations were also found to be expanded and depleted, respectively, in the atlas, their changes were not statistically significant (fdr > 0.05) (Fig. 6b).

Comparison of the atlas-based estimates of differential cell type abundances across tumor grades with the same estimates obtained on individual datasets further highlighted the utility of the integrated atlas to uncover unique associations (Fig. 6c). We found that significant estimates of differential cell type abundance in the atlas were not significant or in discordant directions when tested in individual datasets. For instance, *CD4 Tem* cells were observed to increase with grade in two studies (Wang 2024 and Wu Natgen 2021) but after including all the datasets in the atlas, they did not reach atlas-wide significance. Similarly, *Mast* cells yielded no significant changes in two of the datasets tested (Pal 2021 and Wang 2024), although when all datasets were combined in the atlas it was the cell type most significantly decreasing with grade. Only three datasets were included in this comparison since datasets need to have both high grade (grade 3) and low-grade tumors (grade 1 & 2) to be included in this differential abundance analysis, which further highlights the need for integration so that diverse patient and clinical metadata can be pooled in mega-analysis-based approaches.

### Multi-resolution cell type associations with patient survival

**Figure 7.**
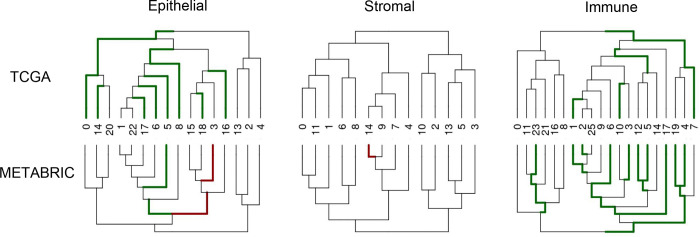
Survival associations of subpopulations found within taxonomic analysis of epithelial, stromal, and immune compartments with respect to TCGA and METABRIC. Green branches indicate a clade that confers a survival advantage (hazard ratio < 1), and red branches indicate a survival disadvantage (hazard ratio > 1) in the respective dataset.

To assess the clinical relevance of the subpopulations identified in the atlas, we performed survival analyses by projecting bulk RNA expression data from both TCGA and METABRIC onto the gene signatures that defined each subgroup within our taxonomic analyses (Fig. 7). To correct for the confounding effects of patient age, proliferation, and inflammation with respect to patient survival ^29,48^, the Cox proportional hazard model also included these factors as covariates (Methods).

Two cell populations were significantly associated with survival in both TCGA and METABRIC and both had a positive survival association (hazard ratio less than 1). These include a subpopulation of epithelial cells (Cluster 5) which is characterized by low stemness (more differentiated) and low grade (Fig 3a,c), and a cluster of *CD8 Tem* immune cells (Cluster 1). Both CD8 effector memory T cell activity and cytotoxicity as well as low grade phenotype within tumors have been previously associated with favorable prognosis in BC ^29,49,50^. Though no single cell type/state was associated with decreased survival in both datasets separately, when combining the results by fisher’s method, increased proliferative stromal cell (*Strom Prolif*.) activity was collectively associated with decreased survival.

## Discussion

In this study we integrated scRNAseq data from 138 primary BC tumors collected across eight studies to create the largest transcriptomic atlas of human BC to date. The large number of patients in our integrated atlas, along with the rigorous annotation of both discrete cell types and continuous cell states, will provide a valuable resource for researchers seeking to explore associations of gene expression and or cell type composition with clinical and molecular phenotypes with increased statistical power.

Our characterization of the cancer epithelial compartment of the atlas revealed substantial heterogeneity between cancer cells along the axes of potency/stemness (differentiation continuum), EMP (epithelial-mesenchymal continuum), expression of hallmark and HBCA pathways, as well as clinical phenotypes. By leveraging K2Taxonomer and random forest models, we performed a multi-resolution characterization of the cancer epithelial compartment and identified the most discriminative factors differentiating epithelial clusters at different levels of the taxonomy of BC epithelial cells. At the first level, we found that BC tumor cells primarily segregated due to EMP, stemness, and basal phenotypes. At the second level, tumor cells primarily segregated by age, suggesting that aging processes can be a major driver of tumor heterogeneity ^51^. At the third level, tumor cells separate through more granular hallmark pathways, tumor subtype and grade. A multi-resolution characterization of cancer cells has several advantages over typical unsupervised clustering approaches including mitigating the risks of over-clustering and thus over-annotation, as well as being able to identify the major factors driving heterogeneity in a dataset.

For the immune and stromal compartments, our analysis reconciled cell type information to established pan-cancer cell types previously characterized, and identified cell states unique to our BC atlas: a collagen expressing Macrophage subtype (*Mac Col.*) and exhausted CD4 cells (*CD4 Ex*), a T cell state associated with the progressive loss of cytokine production and effector function ^52^.

Using these annotations of immune cell type and state, we performed a cell type diversity and differential abundance analysis to capture global and more granular changes in cell type composition within the TME with respect to clinical phenotypes such as tumor subtype (ER+, HER2+, TNBC). Through the cell type diversity analysis, we uncovered associations between increasing tumor grade and decreasing global diversity within the TME, as well as age-associated changes across epithelial, stromal, and immune cells. Through the differential abundance analysis, we resolved differences in immune cell type composition unique to each subtype that provide insight into effective cancer therapies. The expansion of *Mac Lipo* in TNBC is of interest and may help explain recent findings suggesting that TNBC is uniquely susceptible to treatment with lipid-acting drugs ^53^. The expansion of *CD4 Tregs* in HER2+ tumors, may explain the effectiveness of targeted therapies such as Traztuzumab that are known to modulate *Treg* activity ^54^, and highlights the need to identify additional therapies targeting *Treg* activity given that *CD4 Tregs* are known to promote an immunosuppressive TME that can lead to tumor escape ^55^. The enrichment of conventional dendritic cells *cDC* and *Mac Col.* in TNBC and ER+, respectively, also represent promising targets for future therapies given their known role in antigen presentation to activate anti-tumor T cells and in remodeling the extracellular matrix, respectively ^56,57^. Collectively, these subtype specific differences in the immune TME may help explain the effectiveness of existing cancer therapies and prioritize future targets. Further, by repeating this analysis in subsets of the atlas limited to the original datasets, we found that estimates of differential cell type abundance vary considerably between datasets, highlighting the need to pool data to arrive at more robust estimates and to increase the statistical significance of associations found between cell type composition and clinical phenotypes.

Finally, our atlas-wide survival analysis of cell type subpopulations based on bulk data from both TCGA and METABRIC (Supp Table 4) revealed both known associations, such as decreased mortality risk associated with increased CD8 memory T cell activity and low-grade cancer epithelial cells, as well as novel associations, such as increased mortality risk associated with increased proliferative stromal cells (*Strom Prolif.*), which could be prognostic in BC.

One limitation of this atlas is the absence of adipocytes, despite their known importance in cancer progression and aggressiveness ^58^, due to the technical limitations of scRNAseq. Other technologies such as single-nucleus RNA sequencing, and spatial transcriptomics are needed to characterize the heterogeneity in adipocytes across patients. Another limitation is that the atlas represents a static snapshot of the transcriptome and does not capture dynamic changes in response to treatments like radiation or chemotherapy, which could significantly alter the cellular composition and gene expression profiles. Future studies incorporating temporal analyses of cellular responses to various therapies could enhance the atlas’ utility in guiding personalized treatment strategies and understanding mechanisms of treatment resistance.

In summary, this integrated scRNAseq BC atlas, the largest of its kind, fills a crucial need in our understanding of tumor heterogeneity by providing a ‘reference’ landscape of cell populations which not only enables statistically powered analyses of cell type diversity, composition, and survival, but also provides a framework for the construction of further atlases to interrogate tumor heterogeneity.

## Methods

### Data Acquisition

To create an unbiased scRNAseq atlas of human BC, we integrated publicly available datasets that contained untreated samples (samples from patients who had not undergone chemotherapy or other treatment before sequencing) and samples that were not sorted prior to sequencing (not biased towards a particular cell type). Given these criteria, we identified eight datasets to include in this atlas, totaling 138 patients and 621,200 cells.

### Data Preprocessing

Each scRNAseq dataset was first pre-processed using Seurat ^59^ to remove low-quality cells, doublets, and normalize data before integration. We adopted current scRNAseq pre-processing best-practices by using adaptive thresholding based on median absolute deviation, instead of manual cutoffs, to filter out outlier cells based on three criteria: mitochondrial genes expression, number of unique genes detected per cell, and total number of genes detected per cell ^60,61^. For studies that only provided pre-filtered data, we retained all cells for downstream analysis. Following filtering, doublet scores were obtained using scDblFinder ^62^ and expression profiles were normalized by total expression and log-transformed using Seurat’s *LogNormalize* function which has been demonstrated to work well in prior benchmarking ^63^.

### Integration of Single Cell Data

Integration of filtered scRNAseq data was performed using various methods: Seurat’s V5 reciprocal principal component analysis (RPCA) ^59^, fastMNN ^64^, Harmony ^65^, scVI ^66^, and scANVI ^67^. To facilitate fair comparison between the integration methods, the same number of variable features (5000), and PC dimensions (200) were used for integration when possible. For scVI and scANVI, integration is performed at the level of raw counts instead of embeddings, so only the variable feature selection process was included. To avoid biasing the integration towards individual sources of annotations e.g. author annotations or SingleR/CellTypist annotations, only the batch correction metrics: silhouette score, k-nearest neighbor batch effect test (kBET), and integration local inverse Simpson’s index (iLISI), were used to assess integration performance. The best performing integration according to these metrics – Seurat V5’s RPCA, was chosen for downstream analysis (Fig 2a).

### Inference of PAM50 Subtype

To infer molecular subtypes of each sample using the PAM50 method, we first calculated a ‘pseudo-bulk’ profile for each tumor using Seurat’s *AggregateExpression* function, and then applied *molecular.subtyping* from the *genefu* R package to each pseudo-bulk profile with default parameters ^68^.

### Discrete annotation of cell types

Annotation of cell types was performed using a combination of reference based annotation methods: singleR ^30^, CellTypist ^31,69^, unsupervised recursive partitioning: K2Taxonomer ^29^, and unsupervised clustering followed by differential expression analysis: MAST ^32^.

#### Reference-based annotation of cell type

Both singleR and CellTypist require a labelled scRNAseq reference dataset to compare query cells with. Since all of the samples in this study originate from breast tissue, we leveraged labeled scRNAseq data from the healthy breast cell atlas (HBCA) as reference ^10^. For singleR, the wilcox method *de.method = “Wilcox”* was used as this is more appropriate than the default methods when applying singleR to single cell data. For CellTypist, we used a pre-trained breast tissue model that used labeled data from HBCA ^69^, and used majority voting to determine the final cell type label *majority_voting = True, mode = ‘best match’*.

#### Unsupervised clustering-based annotation

Both K2Taxonomer and MAST-based annotation rely on unsupervised clustering which was performed using the Louvain algorithm for three subsets of the atlas separately: epithelial, immune, and stromal, based on expression of canonical markers (Epithelial: *EPCAM, KRT8, KRT 18*; Immune: *PTPRC, CD3D, CD3E, CD68*; Stromal: *COL1A1, COL1A2, ENG*). Performing clustering and annotation within each compartment separately enables the detailed characterization of heterogeneity within each compartment.

To identify differentially expressed genes (DEGs) between clusters, we used Seurat’s *FindAllMarkers* with the parameters *test.use = ‘MAST’*, and *latent.vars = ‘batch’* to account for the negative-binomial distribution of single cell data and to control for dataset-specific effects respectively.

Whilst methods like MAST can quantify differences at the cluster level, the number of clusters, and the resulting DEGs obtained from downstream analysis, relies heavily on arbitrary values of the ‘resolution’ parameter in typical unsupervised clustering algorithms. To mitigate these limitations, we leveraged K2Taxonomer to learn hierarchical relationships between the clusters identified in each compartment, enabling the annotation of clades of clusters instead of just individual clusters. To accomplish this, K2Taxonomer was applied to the integrated embeddings obtained from RPCA, with the following parameters (*featMetric=“F”, nBoots=400, clustFunc=cKmeansDownsampleSqrt*) which are recommended for large scRNAseq datasets ^29^. To estimate the differentially expressed genes (DEGs) between nodes in the dendrogram, we used the normalized gene expression matrix and K2Taxonomer’s built in function *runDGEmods* with batch variable as a covariate to control for dataset-specific effects.

#### Random Forest-based annotation of discriminative factors

The *randomForest* package was used to train random forests to classify cluster membership, or meta-cluster membership (as determined by K2 Taxonomer), of single cell profiles based on expression of continuous scores related to cell stemness, EMP, HBCA cell type, malignancy, and clinical metadata. The model was run with the parameter *importance = TRUE* to extract the most important features (as determined by gini-based importances), that differentiated epithelial clusters from each other.

### Continuous annotation of cell states

Classifying discrete cell types is just one facet of heterogeneity, and there are many transient functional programs or cell states that require a more continuous scoring ^70^. To characterize heterogeneity of functional states within the cancer epithelial compartment of our BC atlas we scored each cell in terms of its malignancy with inferCNV ^27^, stemness with Cytotrace2 ^25^, Epithelial-Mesenchymal plasticity ^26^, expression of hallmark pathways ^28,71^, as well as expression of epithelial subtypes identified in HBCA ^10^.

#### Inference of copy number variations

InferCNV was used to estimate copy number alterations for tumor epithelial cells from each dataset independently with the following parameters: *cutoff = 0.1*, *cluster_by_groups = TRUE*, *denoise = TRUE* and *HMM = FALSE* that are recommended for 10X scRNAseq data. Immune and stromal cells from each dataset were used as normal references, and from these normal reference sets, 100 immune and stromal cells were sampled to act as negative controls to validate inferCNV output.

For each cell, inferCNV estimates a score for each gene that is above one if there is inferred copy number gain, and below one if there is inferred copy number loss. We summarized these scores to estimate a malignancy score per cell that represents the mean absolute CNV score.


$$\mathrm{Malignancy}\;\mathrm{Score}\;=\frac{\sum |\mathrm{CNV}\;\mathrm{score}|}{\#\mathrm{Genes}}$$

To classify each cell as malignant or non-malignant, for each dataset we found the 90% interval of malignancy scores of the negative control cells (non-epithelial cells) and labeled all epithelial cells outside of this interval as malignant (Supp Fig. 6).

#### Scoring of gene set activity

To characterize the heterogeneity of functional states present in the atlas, we leveraged publicly available transcriptional signatures (Supp Table 4) and used a rank-based method (AUCell) ^72^, with default parameters, to score enrichment of signatures in each cell. For the immune compartment, we scored cells using signatures from pan-cancer studies of myeloid and lymphoid cell states ^33,35^, as well as signatures of immune cell types from HBCA ^10^. For the stromal compartment, we scored cells using signatures from a pan-cancer study of stromal cell heterogeneity ^38^. For the epithelial compartment, we scored cells using canonical hallmark signatures ^28^, as well as signatures of epithelial cell types from HBCA ^10^.

### Differential Diversity and Abundance Analysis

#### Cell Type Diversity Analysis

We used an entropy-based cell type diversity metric (CTDS) ^40^ to assess how the overall diversity in a patient’s TME changes across subtype, age, and tumor grade. The CTDS metric accounts for the number of cells per sample and the compositional nature of proportional data which enables comparison across samples.

#### Differential Abundance Analysis

To test differences in cell type proportions across different phenotype groups e.g. tumor grade and subtype, we used the R package *sccomp* with *bimodal_mean_variability_association = TRUE* which is recommended for scRNAseq data ^41^. To isolate the effects of tumor grade on TME composition, we controlled for tumor subtype, age, and batch with the following formula:

$${\mathrm{celltype}}_{\mathrm{count}\;}∼\mathrm{grade}\;+\;\mathrm{pam}50+\;\mathrm{age}\;+(1|\mathrm{batch})$$

Similarly, to isolate the effects of tumor subtype on TME composition, we controlled for grade, age, and batch.


$${\mathrm{celltype}}_{\mathrm{count}\;}∼(\mathrm{LumA}/\mathrm{LumB}/\mathrm{Her}2/\mathrm{Normal\_like}\;)+\;\mathrm{grade}\;+\;\mathrm{age}\;+(1|\mathrm{batch})$$

### Multi-resolution survival analysis of cell type populations

To identify the clinical relevance of cell type annotations identified in the atlas, we extracted differentially expressed genes defining each subpopulation found in our taxonomic analysis of each compartment (K2Taxonomer) ^29^, and used the R package *brcasurv* ^73^ to model the association between the expression of subpopulation specific genes and patient survival in both TCGA ^3^ and METABRIC ^74^.

To address possible confounders in the survival analysis, we included patients age, as well as patient-level gene set projection scores of inflammation and proliferation that have previously been associated with poor prognosis and demonstrated to be necessary for correction in survival modelling ^29,48,75^. The patient-level gene set scores were obtained by using the R package *GSVA* ^76^ to project inflammation ^75^, proliferation ^48^, as well as the cell type specific gene sets onto transcriptional profiles from TCGA and METABRIC. The Cox proportional hazards model underlying the survival analysis controlled for these confounders with the following formula:

$$Surv(\mathrm{patient})∼{\;\mathrm{celltype}}_{\mathrm{gsva}\;}+\;\mathrm{age}\;+\;{\mathrm{inflam}}_{\mathrm{gsva}}\;+\;{\mathrm{prolif}}_{\mathrm{gsva}\;}$$

To control for multiple testing, we corrected p-values in each cox model using the FDR method ^77^ in the *p.adjust* R package, and to reconcile p-values between analyses performed in TCGA and METABRIC, we used the Fisher’s method for combining p-values ^78^.

## Supplementary Material

Supplement 1

Supplement 2

Supplement 3

Supplement 4

Supplement 5

## Figures and Tables

**Table 1:** Summary of Datasets used in Atlas

Dataset	# Patients	# Cells	System	PMID	Data Link

Pal et al. 2021	33	224,823	10× 3’	33950524	GSE161529
Bassez et al. 2021	31	78,549	10× 5’	33958794	Lambrechts lab
Wu et al. 2021	26	99,876	10× 3’ v2	34493872	GSE176078
Wang et al. 2024	14	48,553	10× 3’ v2	38382468	GSE248288
Qian et al. 2020	14	40,665	10× 3’ v2 & 10× 5’ v2	32561858	Lambrechts lab
Tietscher et al. 2023	12	97,301	10× 3’ v3	36609566	E-MTAB-10607
Liu et al. 2023	4	27,490	10× 3’ v3	36594618	GSE225600
Gao et al. 2021	4	3,943	10× 3’ v2 & 10× 5’ v2	33462507	GSE148673
**Total**	**138**	**621,200**			

## Data Availability

All code is available at: https://github.com/montilab/brca_atlas and figshare https://doi.org/10.6084/m9.figshare.28685012.v1.
