# Supplementary material for: A highly resolved integrated transcriptomic atlas of human breast cancers
Source: bioRxiv. 2025 May 3:2025.03.13.643025. Originally published 2025 Mar 15. Preprint. [Version 2] doi: 10.1101/2025.03.13.643025 (PMC11952505; doi:10.1101/2025.03.13.643025)
Supplement: Supplement 5 [file NIHPP2025.03.13.643025v2-supplement-5.pdf]

bioRxiv preprint doi: https://doi.org/10.1101/2025.03.13.643025; this version posted May 3, 2025. The copyright holder for this preprint (which was not certified by peer review) is the author/funder, who has granted bioRxiv a license to display the preprint in perpetuity. It is made available under aCC-BY 4.0 International license.

Supplementary Figure 1. UMAP of unintegrated data colored by dataset, subtype, grade, cell typist, singleR, and author annotation.

Supplementary Figure 2. UMAP of RPCA integrated data colored by clustering, and broad cell type annotations from singleR, CellTypist, and author provided metadata.

Supplementary Figure 3. Cancer Epithelial Compartment Diversity. A) UMAP of cancer epithelial cells colored by clustering performed at 0.2 resolution. B) UMAP of cancer epithelial cells colored by HBCA annotation from CellTypist. C) UMAP of cancer epithelial cells colored by tumor subtype. D) UMAP of cancer epithelial cells colored by PAM 50 subtype. E) Distribution of patients across each of the clusters. F) Entropy of donor proportions across each of the clusters.

bioRxiv preprint doi: https://doi.org/10.1101/2025.03.13.643025; this version posted May 3, 2025. The copyright holder for this preprint (which was not certified by peer review) is the author/funder, who has granted bioRxiv a license to display the preprint in perpetuity. It is made available under aCC-BY 4.0 International license.

Supplementary Figure 4. UMAP of immune compartment colored by clustering, and cell type annotations from singleR, CellTypist, and author provided metadata.

Supplementary Figure 5. UMAP of stromal compartment colored by clustering, and cell type annotations from singleR, CellTypist, and author provided metadata.

Supplementary Figure 6. Distribution of inferCNV scores for each of the datasets included in the atlas. Immune and stromal cells sampled from each donor are in yellow whereas epithelial cells are in orange. The empirical 90% interval of malignancy scores for normal cells is highlighted for each dataset and is the interval used to classify epithelial cells as malignant or non-malignant.

S1

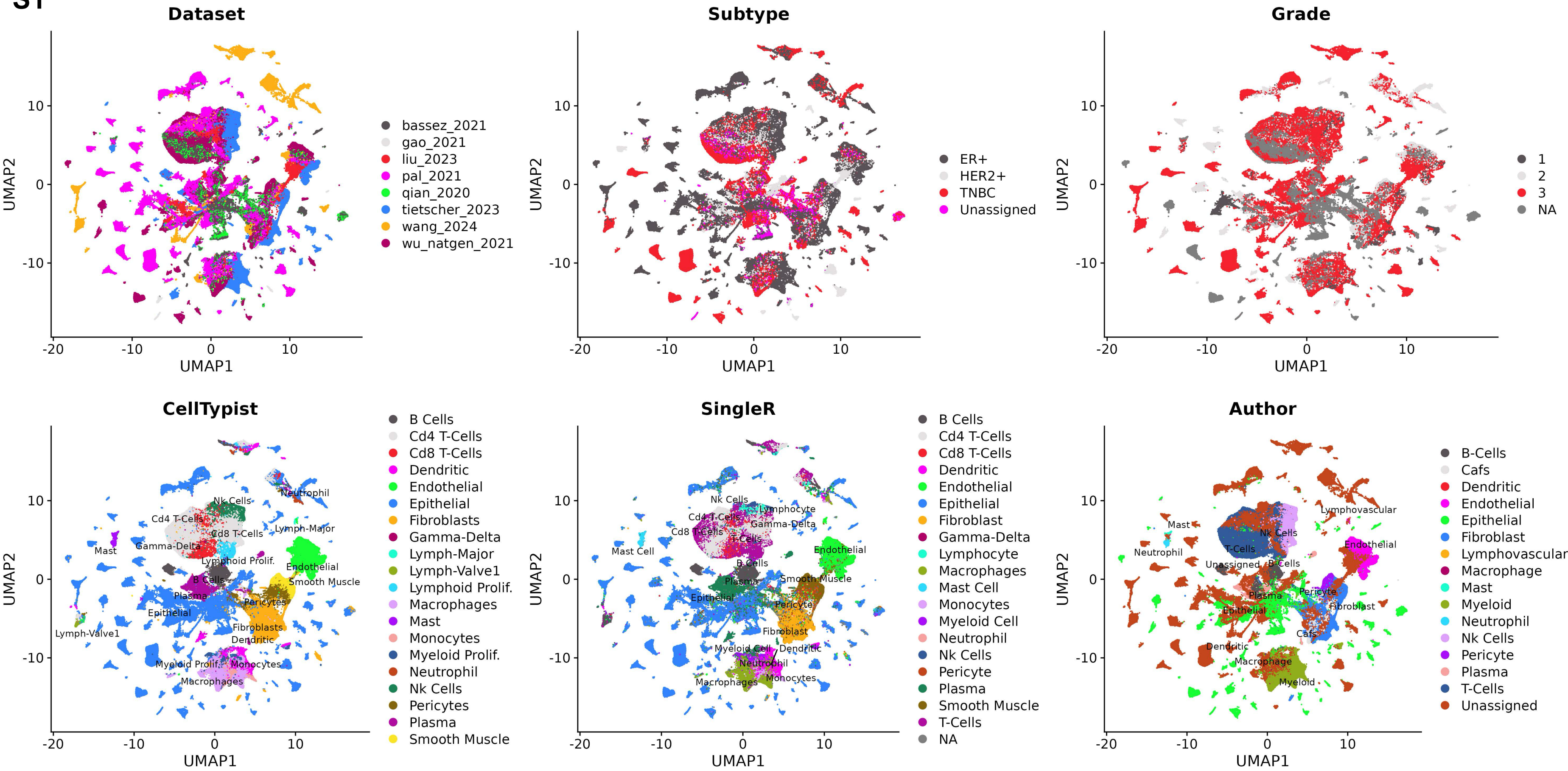
Dataset
Subtype
Grade
UMAP1
UMAP2
bassez_2021
gao_2021
liu_2023
pal_2021
qian_2020
tietscher_2023
wang_2024
wu_natgen_2021
ER+
HER2+
TNBC
Unassigned
1
2
3
NA
CellTypist
SingleR
Author
B Cells
Cd4 T-Cells
Cd8 T-Cells
Dendritic
Endothelial
Epithelial
Fibroblasts
Gamma-Delta
Lymph-Major
Lymph-Valve1
Lymphoid Prolif.
Macrophages
Mast
Monocytes
Myeloid Prolif.
Neutrophil
Nk Cells
Pericytes
Plasma
Smooth Muscle
B Cells
Cd4 T-Cells
Cd8 T-Cells
Dendritic
Endothelial
Epithelial
Fibroblast
Gamma-Delta
Lymphocyte
Macrophages
Mast Cell
Monocytes
Myeloid Cell
Neutrophil
Nk Cells
Pericyte
Plasma
Smooth Muscle
T-Cells
NA
B-Cells
Cafs
Dendritic
Endothelial
Epithelial
Fibroblast
Lymphovascular
Macrophage
Mast
Myeloid
Neutrophil
Nk Cells
Pericyte
Plasma
T-Cells
Unassigned

S2

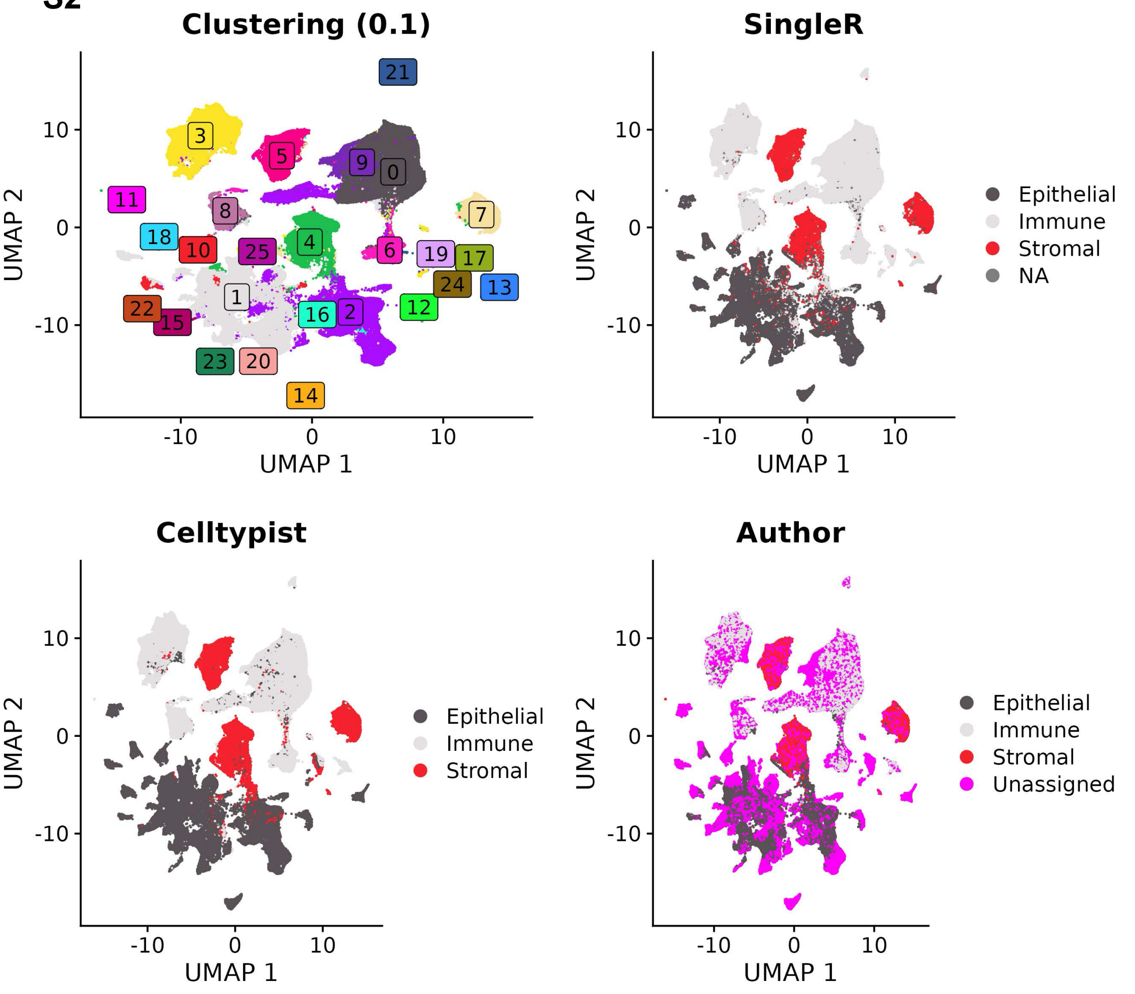
Clustering (0.1)
SingleR
Celltypist
Author
UMAP 1
UMAP 2
Epithelial
Immune
Stromal
NA
Unassigned

S3

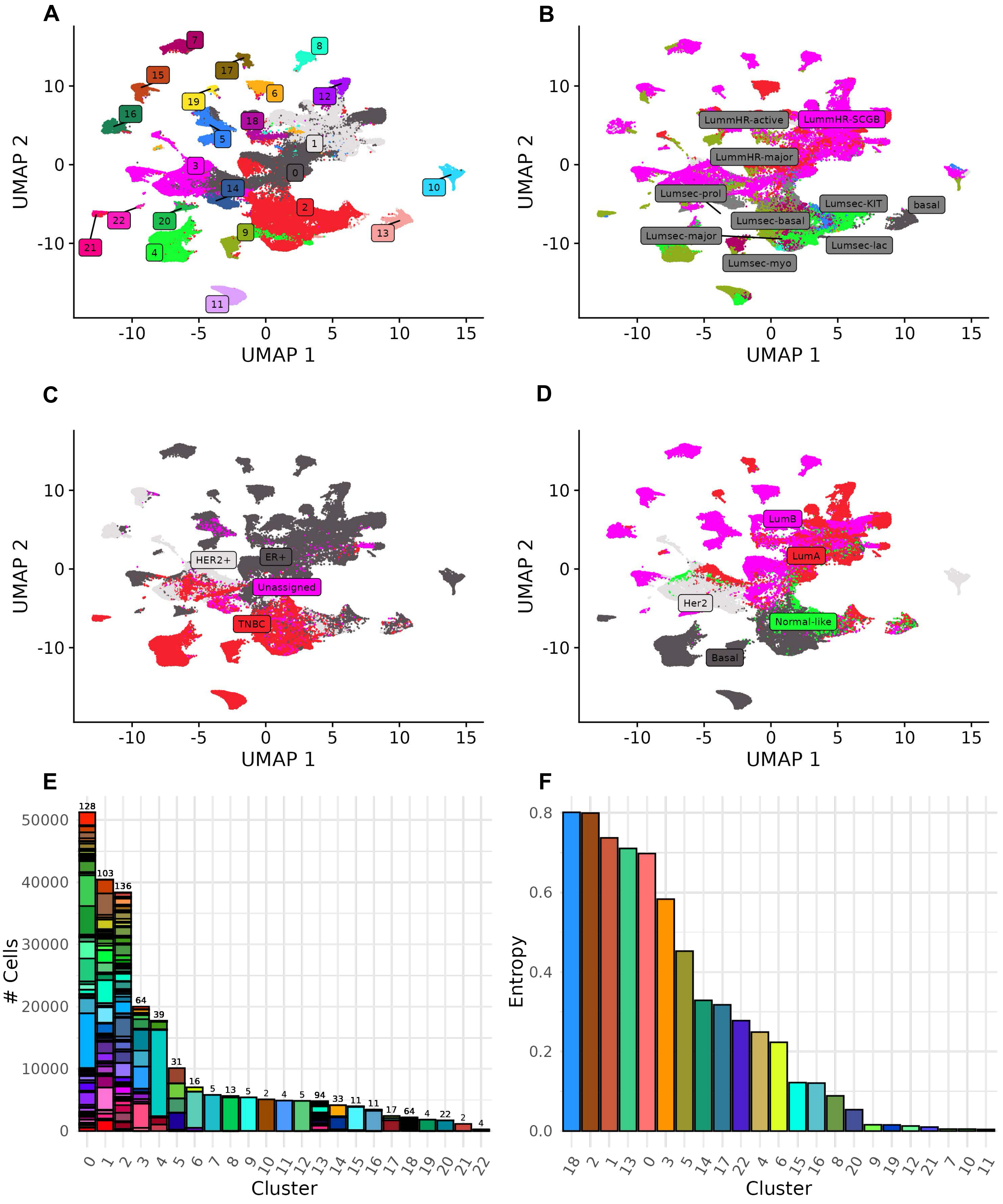
A
B
C
D
E
F
UMAP 1
UMAP 2
LummHR-active
LummHR-SCGB
LummHR-major
Lumsec-prol
Lumsec-KIT
basal
Lumsec-basal
Lumsec-major
Lumsec-lac
Lumsec-myo
HER2+
ER+
Unassigned
TNBC
LumB
LumA
Her2
Normal-like
Basal
Cells
Cluster
Entropy

S4

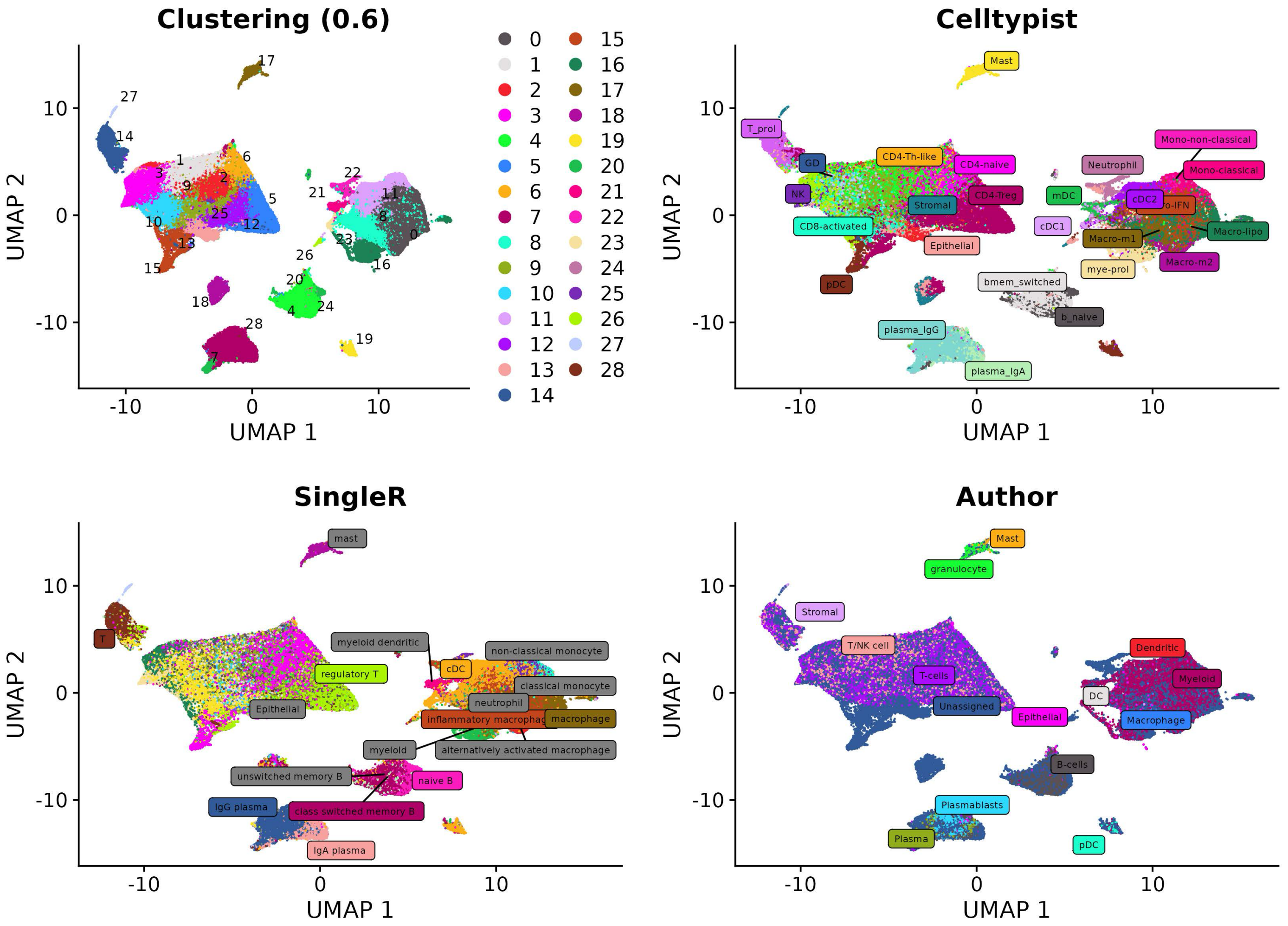

Clustering (0.6)
Celltypist
SingleR
Author
UMAP 1
UMAP 2
Mast
T_prol
GD
NK
CD4-Th-like
CD4-naive
CD4-Treg
Stromal
CD8-activated
Epithelial
pDC
Neutrophil
Mono-non-classical
Mono-classical
mDC
cDC2
cDC1
Macro-m1
Macro-lipo
Macro-m2
mye-prol
bmem_switched
b_naive
plasma_IgG
plasma_IgA
mast
T
myeloid dendritic
non-classical monocyte
cDC
regulatory T
classical monocyte
neutrophil
Epithelial
inflammatory macrophage
macrophage
myeloid
alternatively activated macrophage
unswitched memory B
naive B
IgG plasma
class switched memory B
IgA plasma
granulocyte
Stromal
T/NK cell
T-cells
Unassigned
Epithelial
Dendritic
Myeloid
DC
Macrophage
B-cells
Plasmablasts
Plasma
pDC

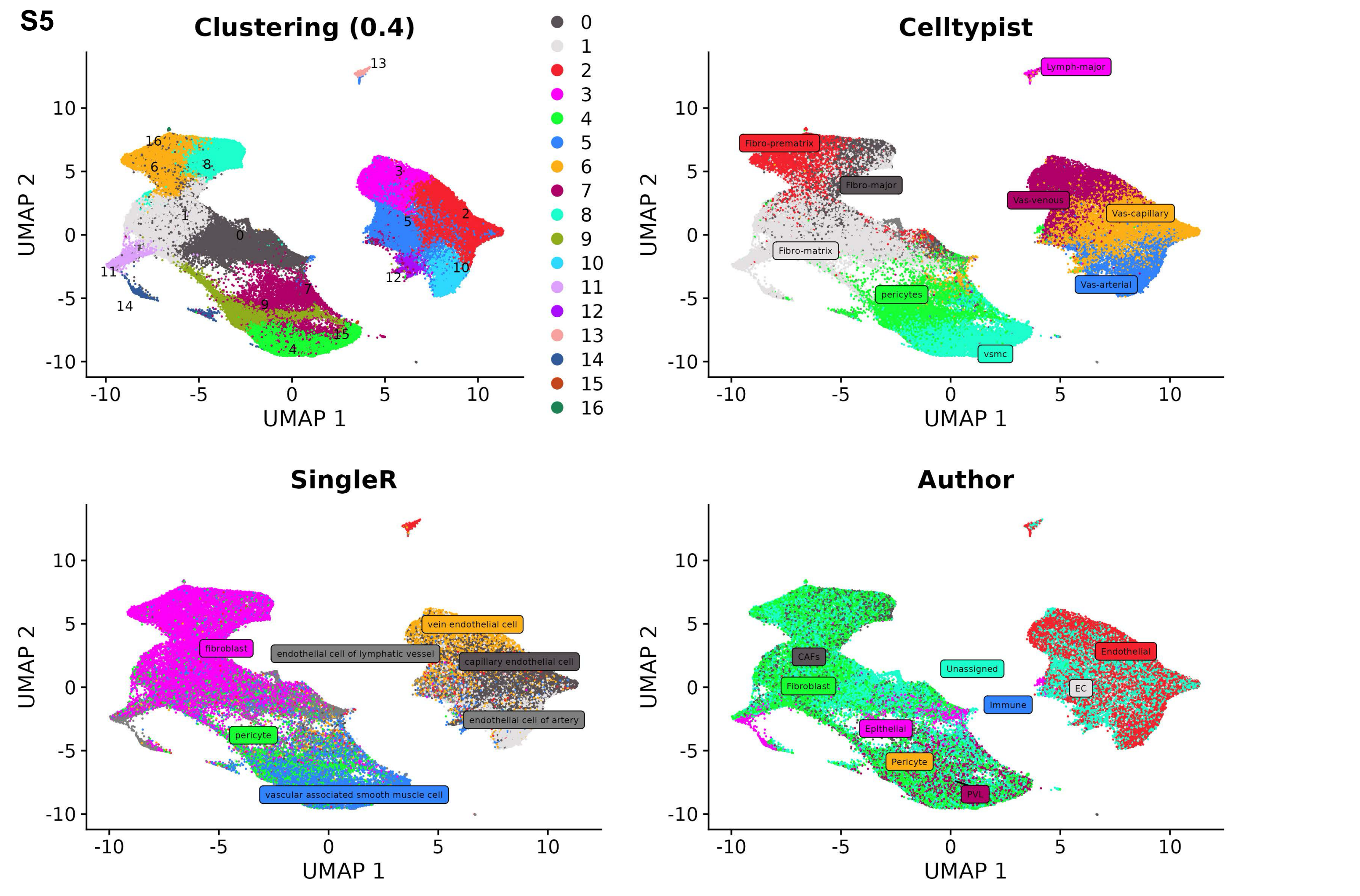
S5
Clustering (0.4)
Celltypist
SingleR
Author
UMAP 1
UMAP 2
0
1
2
3
4
5
6
7
8
9
10
11
12
13
14
15
16
Lymph-major
Fibro-prematrix
Fibro-major
Vas-venous
Vas-capillary
Fibro-matrix
Vas-arterial
pericytes
vsmc
vein endothelial cell
fibroblast
endothelial cell of lymphatic vessel
capillary endothelial cell
endothelial cell of artery
pericyte
vascular associated smooth muscle cell
Endothelial
CAFs
Unassigned
Fibroblast
EC
Immune
Epithelial
Pericyte
PVL

S6

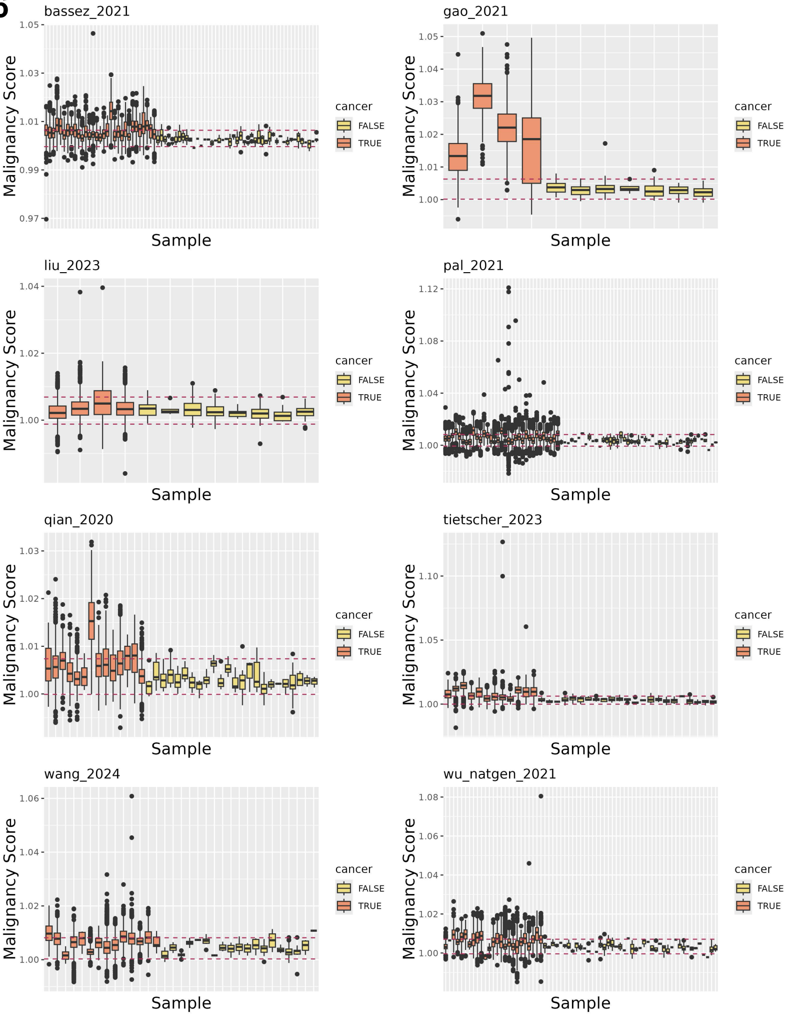
bassez_2021
Malignancy Score
1.05
1.03
1.01
0.99
0.97
Sample
cancer
FALSE
TRUE
gao_2021
Malignancy Score
1.05
1.04
1.03
1.02
1.01
1.00
Sample
cancer
FALSE
TRUE
liu_2023
Malignancy Score
1.04
1.02
1.00
Sample
cancer
FALSE
TRUE
pal_2021
Malignancy Score
1.12
1.08
1.04
1.00
Sample
cancer
FALSE
TRUE
qian_2020
Malignancy Score
1.03
1.02
1.01
1.00
Sample
cancer
FALSE
TRUE
tietscher_2023
Malignancy Score
1.10
1.05
1.00
Sample
cancer
FALSE
TRUE
wang_2024
Malignancy Score
1.06
1.04
1.02
1.00
Sample
cancer
FALSE
TRUE
wu_natgen_2021
Malignancy Score
1.08
1.06
1.04
1.02
1.00
Sample
cancer
FALSE
TRUE
