# Supplementary material for: A highly resolved integrated transcriptomic atlas of human breast cancers
Source: bioRxiv. 2025 Mar 15:2025.03.13.643025. Preprint. [Version 1] doi: 10.1101/2025.03.13.643025 (PMC11952505; doi:10.1101/2025.03.13.643025)
Supplement: Supplementary file 1 [file NIHPP2025.03.13.643025v1-supplement-5.pdf]

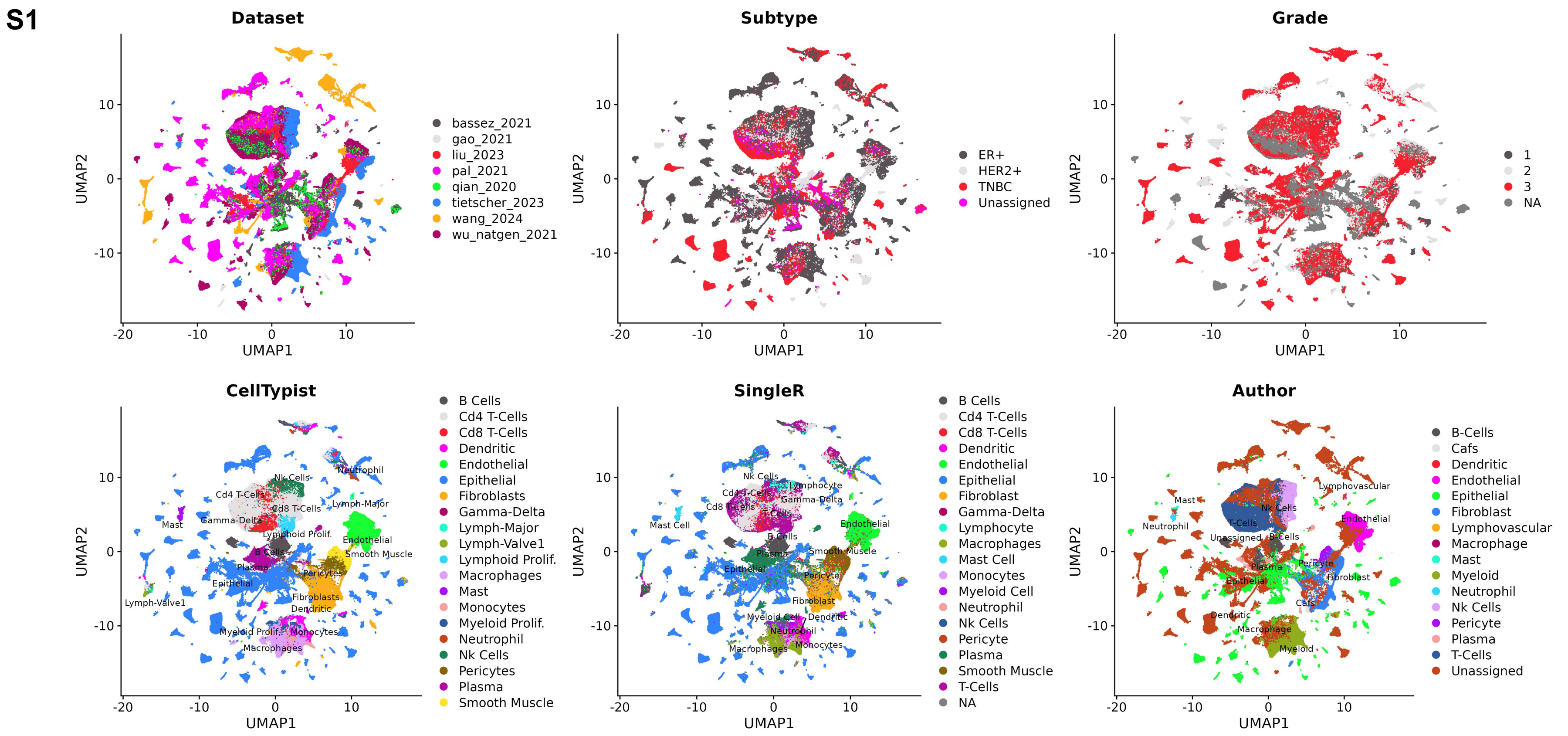

Supplementary Figure 1. UMAP of unintegrated data colored by dataset, subtype, grade, celltypist, singleR, and author annotation.

S2

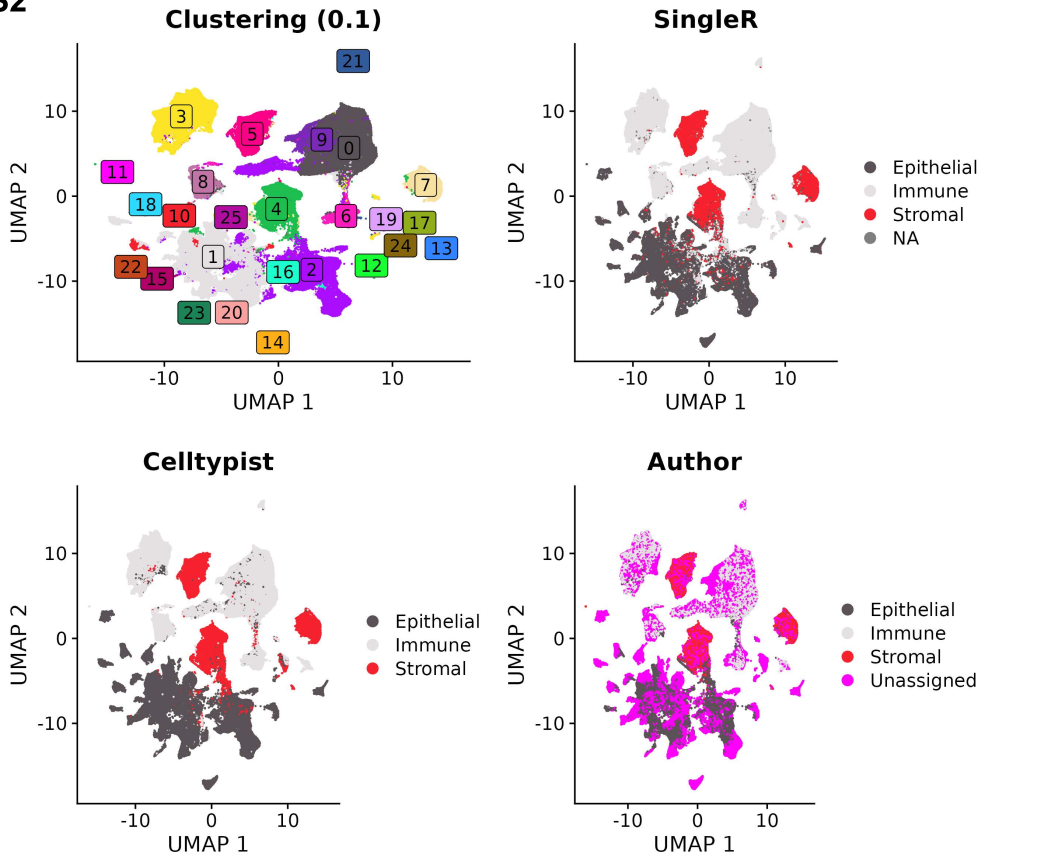

Supplementary Figure 2. UMAP of RPCA integrated data colored by clustering, and broad cell type annotations from singleR, CellTypist, and author provided metadata.

S3

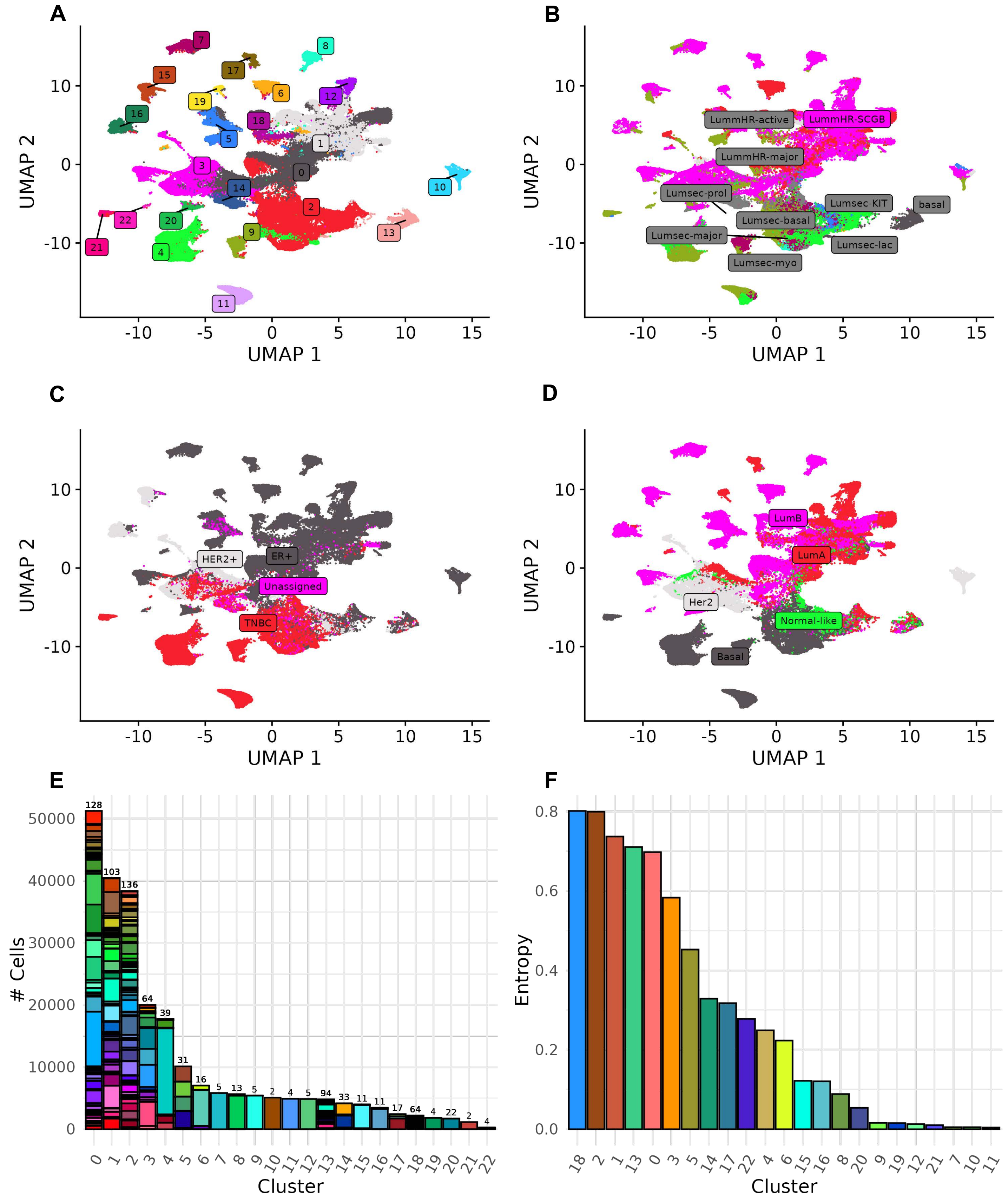

Supplementary Figure 3. Cancer Epithelial Compartment Diversity. A) UMAP of cancer epithelial cells colored by clustering performed at 0.2 resolution. B) UMAP of cancer epithelial cells colored by HBCA annotation from CellTypist. C) UMAP of cancer epithelial cells colored by tumor subtype. D) UMAP of cancer epithelial cells 625 colored by PAM 50 subtype. E) Distribution of patients across each of the clusters. F) Entropy of donor proportions across each of the clusters.

S4

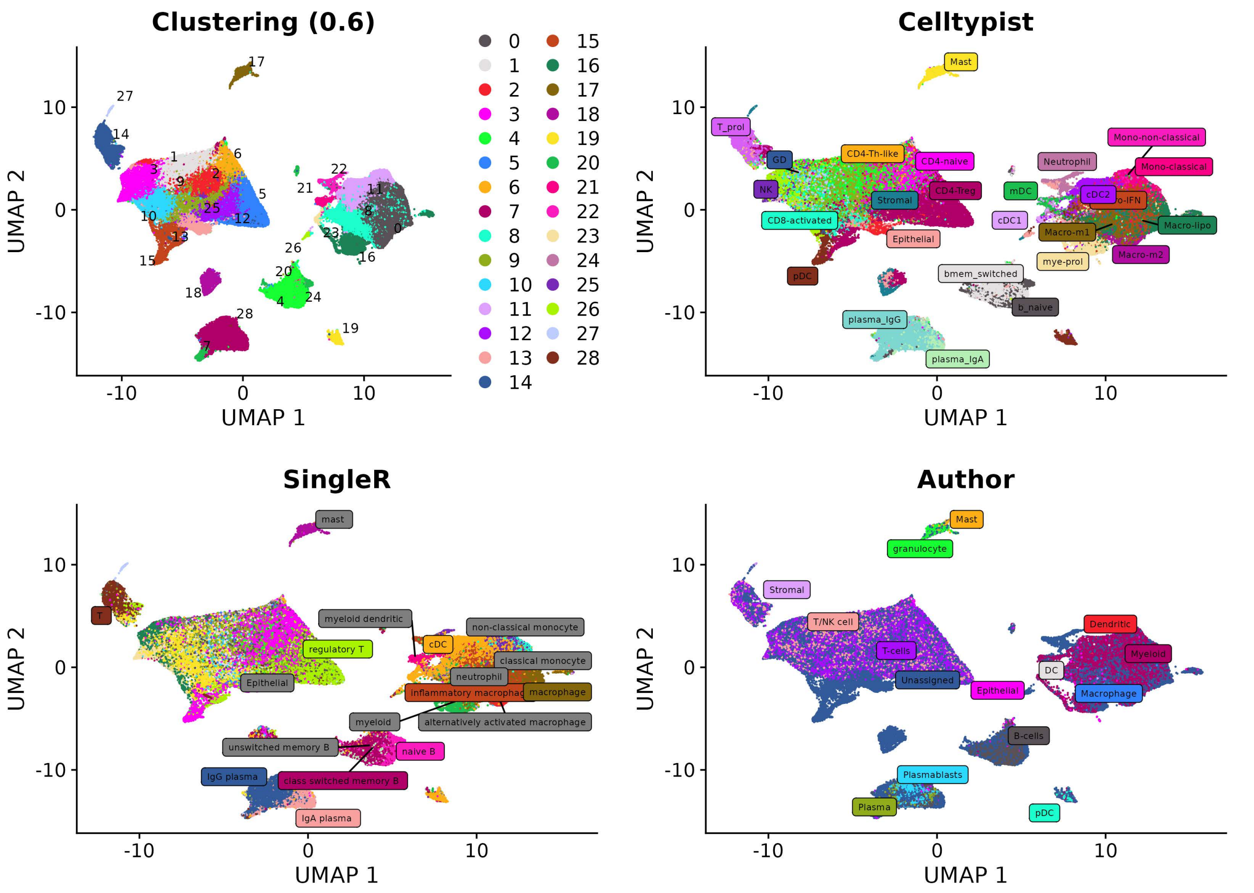

Supplementary Figure 4. UMAP of immune compartment colored by clustering, and cell type annotations from singleR, CellTypist, and author provided metadata.

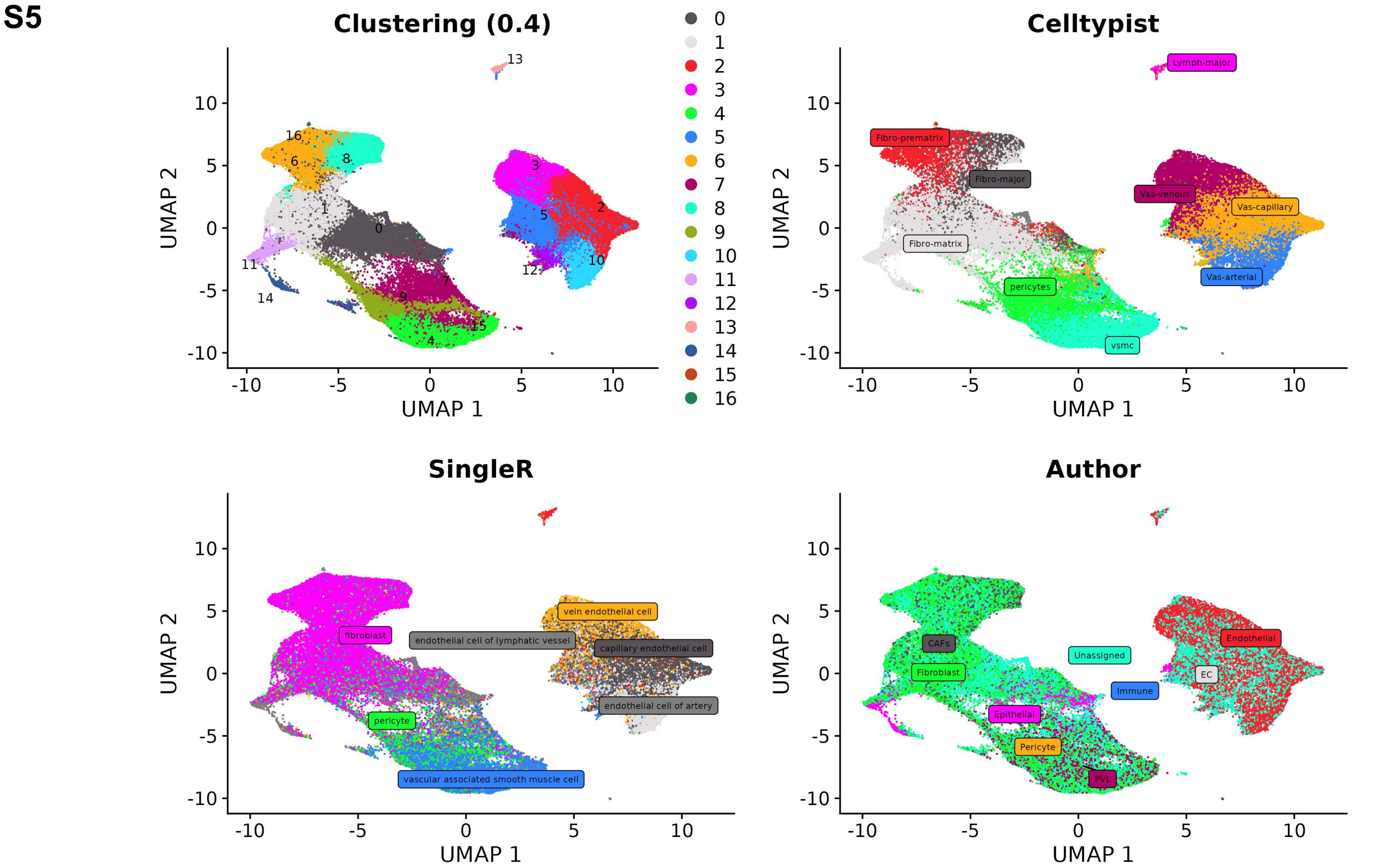

Supplementary Figure 5. UMAP of stromal compartment colored by clustering, and cell type annotations from singleR, CellTypist, and author provided metadata.

bioRxiv preprint doi: https://doi.org/10.1101/2025.03.13.643025; this version posted March 15, 2025. The copyright holder for this preprint (which was not certified by peer review) is the author/funder, who has granted bioRxiv a license to display the preprint in perpetuity. It is made available under aCC-BY 4.0 International license.

S6

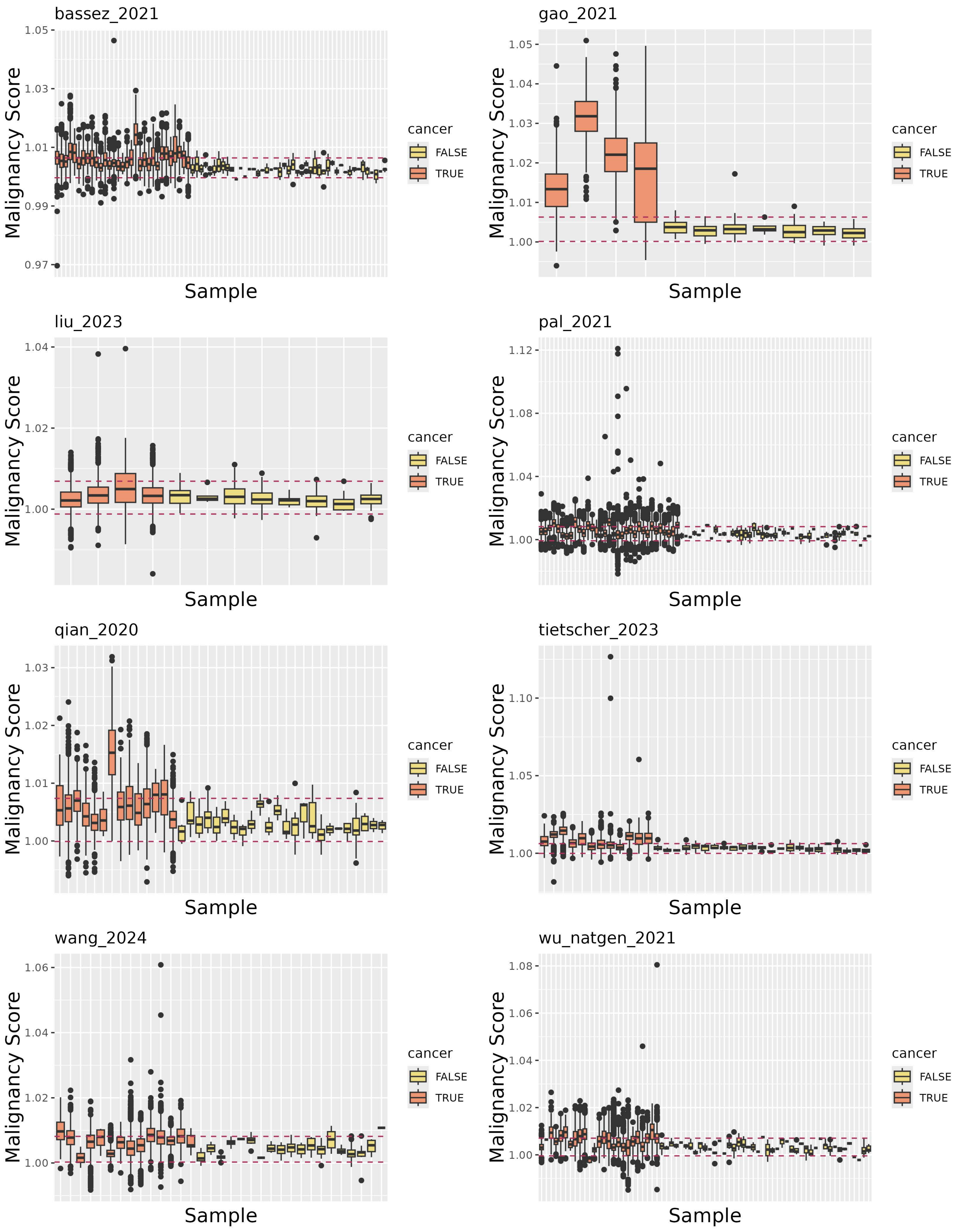

Supplementary Figure 6. Distribution of inferCNV scores for each of the datasets included in the atlas. Immune and stromal cells sampled from each donor are in yellow whereas epithelial cells are in orange. The empirical 90% interval of malignancy scores for normal cells is highlighted for each dataset and is the interval used to classify epithelial cells as malignant or non-malignant.
